# Auditory spatial attention is encoded in a retinotopic reference frame across eye-movements

**DOI:** 10.1371/journal.pone.0202414

**Published:** 2018-08-20

**Authors:** Martijn Jan Schut, Nathan Van der Stoep, Stefan Van der Stigchel

**Affiliations:** Experimental Psychology, Helmholtz Institute, Utrecht University, Utrecht, The Netherlands; University of Muenster, GERMANY

## Abstract

The retinal location of visual information changes each time we move our eyes. Although it is now known that visual information is remapped in retinotopic coordinates across eye-movements (saccades), it is currently unclear how head-centered auditory information is remapped across saccades. Keeping track of the location of a sound source in retinotopic coordinates requires a rapid multi-modal reference frame transformation when making saccades. To reveal this reference frame transformation, we designed an experiment where participants attended an auditory or visual cue and executed a saccade. After the saccade had landed, an auditory or visual target could be presented either at the prior retinotopic location or at an uncued location. We observed that both auditory and visual targets presented at prior retinotopic locations were reacted to faster than targets at other locations. In a second experiment, we observed that spatial attention pointers obtained via audition are available in retinotopic coordinates immediately after an eye-movement is made. In a third experiment, we found evidence for an asymmetric cross-modal facilitation of information that is presented at the retinotopic location. In line with prior single cell recording studies, this study provides the first behavioral evidence for immediate auditory and cross-modal transsaccadic updating of spatial attention. These results indicate that our brain has efficient solutions for solving the challenges in localizing sensory input that arise in a dynamic context.

## Introduction

Introspectively, determining that visual input matches auditory input appears trivial. Yet, comparing the location of sensory input between senses is complicated, because locations are encoded in different reference frames, native to their sensory modality. Within midbrain structures, the locations of visual input are encoded relative to the retina (retinotopic, also oculocentric), whereas auditory locations are initially encoded relative to the head [1–6]. Additionally, animal studies have shown that auditory coordinates are also (partially) converted into the dominant visual reference frame (i.e. a retinotopic coordinate space map), in structures such as the superior colliculus [7–11]. Adding to this complexity, each eye-movement shifts the retinotopic location of visual input [12,13]. Humans make several eye-movements per second, requiring auditory spatial information to be represented in retinotopic coordinates immediately after an eye-movement to allow for comparison of auditory and visual locations. Key papers have shown that gaze/eye position affects auditory localization 1) by shifting the localization of auditory stimuli towards the gaze position, and 2) by improving localization of auditory stimuli at the gaze position [14–16]. Yet, how eye-movements, and the introduction of disparity between the retinotopic and craniotopic reference frame, may cause auditory spatial attentional pointers to update is less clear.

Previous studies have shown that observers continue to transiently sample visual information at previous retinotopic locations (retinotopic trace) for a brief period after an eye-movement has been made, due to retinotopic encoding and lingering of visual attention [17,18]. Compared to stimuli at non-retinotopically matched locations, stimuli at the location of the retinotopic trace (1) are responded to faster [17,19], (2) elicit an enhanced P1 and anterior N1 event related potential component [20,21], and (3) correlate with differential blood oxygen level-dependent response patterns in primary visual cortex (V1) and further in the visual processing hierarchy (V4) [21]. These findings (both behavioral and neurophysiological) show that visual attention is retinotopically encoded, and that visual attention lingers in retinotopic coordinates after an eye-movement.

The behavioral effects of the retinotopic lingering of visual attention can be observed in a task originally described by Golomb and colleagues [17]. In this paradigm, participants are instructed to remember the exact location of a stimulus (the memory cue). After this, participants are cued to make a saccade. After the saccade is executed, an oriented bar (probe) is presented at either the previous retinotopic location, the spatiotopic location, or a different (neutral) location after a short or a long delay between saccade offset and probe (target) onset. The authors observed shorter reaction times to the probe when presented at the previous retinotopic location shortly after the saccade, compared to probes presented at other locations shortly after the saccade. The authors attribute this facilitation effect to lingering of visual attention at the location of the retinotopic trace. This attentional lingering at previous retinotopic locations diminished for probes presented later after the saccade, reflecting the decay of the retinotopic trace. In contrast, reaction times to probes at spatiotopic locations are facilitated at longer delays. The authors argue that spatiotopic attentional facilitation with longer delays is the result of attentional updating from the prior retinotopic location to the new spatiotopic location. In conclusion, visual spatial attention is natively encoded in a retinotopic reference frame.

If auditory locations are also encoded in retinotopic coordinates, attentional lingering at the location of the retinotopic trace after a saccade should be observed after both auditory and visual stimulation. Furthermore, it is currently unclear whether the time course of the decay of attention at the location of the retinotopic trace is similar for audition and vision. If the magnitude and the time course of attentional facilitation of the retinotopic trace is similar across modalities, it would suggest that visual and auditory attention are affected by a shared attentional updating process.

The experiments by Golomb and colleagues provide evidence for a dual-process model of attentional updating [17,22]. In this model, visual attention lingers in retinotopic coordinates immediately after a saccade, and visual attention is simultaneously updated to the new spatiotopic location. The observed attentional facilitation at spatiotopic locations increases with longer delays, whilst attentional facilitation decreases at the retinotopic location with longer delays. Interestingly, spatiotopic updating is slower when less visual information is available, whereas retinotopic lingering is unaffected by the amount of visual input [23]. In the current study, the visual-only task will have the fixation point, memory cues, and probes as visual anchors. The auditory-only task will only have the fixation point as a visual anchor. We expected this reduction of visual anchors to slow spatiotopic updating relative to the visual-only condition, perhaps halting it altogether.

**Fig 1:**
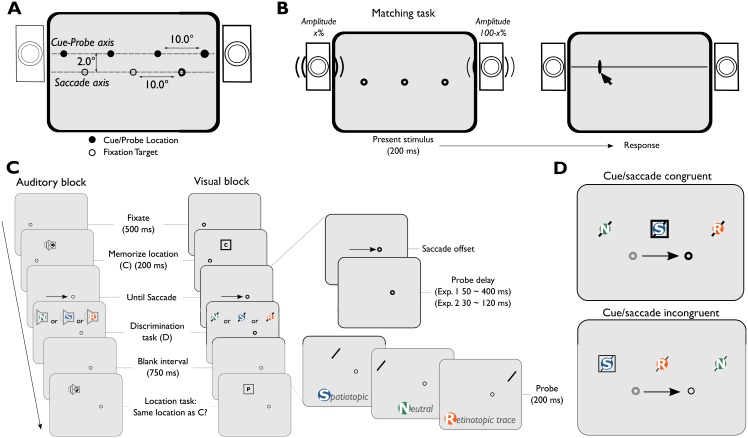
Experimental procedure for Experiment 1 and 2. A) An illustration of the set-up. Stimuli could appear on two axes on the screen. Fixation targets were presented on the bottom axis, cues and probes on the upper axis. B) A trial in the matching task. White noise was presented for 200 ms, participants clicked where they heard the sound originating from. C) Experimental procedure during the auditory and visual blocks. The discrimination task portion of the trial is shown in further detail on the right. There were different probe locations and probe delays. D) An example of congruent and incongruent probe and memory locations with respect to saccade direction. Square = visual cue location, diagonal line = visual probe location.

In sum, we expected spatiotopic updating for visual stimuli, and slower spatiotopic updating for auditory stimuli. We expect both auditory and visual information to be represented in retinotopic coordinates immediately after the saccade. To investigate whether auditory spatial attention is immediately available in retinotopic coordinates across eye-movements, we adapted the design used by Golomb [17], and created an auditory analogue to the visual task (see Fig 1).

## Methods

### Subjects

Subjects reported normal or corrected-to-normal vision and hearing (Experiment 1: *N* = 17, 12 Female, M_age_ = 23.4 years, Experiment 2: *N* = 17, 9 Female, *M*_*age*_ = 22.2 years). Participants were compensated with €14,- for two hours. Written informed consent was obtained from all participants. All experiments were approved by the faculty ethics committee of Utrecht University (FETC) and in accordance with the Declaration of Helsinki.

### Setup

The experiment was conducted in a darkened, sound-attenuated lab. Participants were seated with their head supported in a chin rest 70 cm from an Asus ROG Swift PG278Q monitor (60.1x34.0 cm, 2560x1440 pixels, 100 Hz). Auditory stimuli were presented with two speakers (Harman/Kardon HK206, frequency response: 90–20,000 Hz), placed along the vertical edges of the screen. The speaker cones were placed 2° above the horizontal meridian of the screen (i.e. at the cue-probe axis), 60° apart horizontally (see Fig 1A). Eye movements were recorded with an EyeLink 1000 (SR Research Ltd., Canada), calibrated with the native 9-point calibration procedure, recording the left eye at 1000 Hz.

### Procedure

The experiments were divided into a matching task (100 trials; Fig 1B), a visual block, and an auditory block (each 240 trials, Fig 1C). The matching task was completed first. Half of the participants completed the visual block before the auditory block, for the remaining participants this order was reversed. Participants completed 25 practice trials before starting each block.

#### Matching task

In the matching task, participants were shown three fixation stimuli while fixating the center. After a 200 ms delay, white noise was played for 200 ms. The stimuli were linearly panned in amplitude in 100 equal steps (from -1; amplitude left = 100%, amplitude right = 0%; to 1), one step per trial in random order (counter-balanced). After the stimulus was presented, participants positioned the mouse cursor where they perceived the sound originating from using a computer mouse. Each participant completed 100 trials in the Matching task. The data for the matching task was used to fit a sigmoid relation between the recorded horizontal location of the reticule (in visual angle, -30° to 30°) and the panning values (-1 to 1). This was done to match stimulus locations in the visual block and the auditory block. In a pilot study, we found that the mapping of panning values with blue noise and pink noise into degrees of visual angle matched the mapping of white noise. For this reason, we only mapped out white noise in the current set of experiments.

#### Visual/Auditory block

The visual and auditory block were kept similar. Participants were instructed to perform two tasks within a trial. To direct spatial attention to a location, participants were tasked with remembering a location of a square (memory cue) presented 5° to the left or right of fixation. The first memory cue was presented for 200 ms, followed by a 200 ms blank screen. The memory cue was either a white box (visual) or a white noise burst (auditory). Next, the fixation point moved from the center of the screen 10° to the left or right. Participants were instructed to make an eye-movement to this location. The memory cue location was non-predictive with respect to saccade direction (Fig 1D). Crucially, after saccade landing a probe was shown. We manipulated (1) the time between saccade offset and probe onset (determined online, Experiment 1: 50 to 400 ms in steps of 50 ms, Experiment 2: 30 to 120 ms in steps of 30 ms), and (2) the location of the probe for the discrimination task. The probe was presented at one of three locations: the same location as the memory cue (Spatiotopic), the pre-saccadic retinotopic location of the memory cue (Retinotopic trace), or an uncued location (Neutral). Participants were instructed to report the identity of the probe as quickly as possible (visual: left/right tilt, auditory: blue noise/pink noise) using the up and down arrow keys. After 1000 ms had passed, a second memory cue was presented at either the same location (as the first cue) or a different location (first cue location ± horizontal offset). Other stimulus parameters of the second memory cue were identical to the first memory cue. Participants responded whether the second memory cue was presented at the same or different location as the first memory cue (‘S’ or ‘D’ key, unspeeded response). During the experiment, the horizontal offset was adapted with a three up, one down staircase to keep the task challenging. This task was implemented to keep the location of the memory cue relevant across eye-movements.

### Stimuli

In all blocks, small white annuli of 0.5° (4.2 cd/m2) were used as fixation stimuli. Fixation stimuli were always presented on an imaginary horizontal line (the Saccade axis, see Fig 1A) placed on the vertical center of the screen. In the visual block, probes were grey bars (12.1 cd/m^2^, 0.1° x 1.0°) that were tilted 45 degrees to the left or right and memory cues were grey squares of 1.0° x 1.0° (7.6 cd/m^2^). Auditory stimuli were panned in amplitude between the two speakers, varying from -1 (left speaker amplitude = amplitude x 1, right speaker amplitude = amplitude x 0) to 1, to create the perception of different sound sources in between the speakers. The auditory stimuli consisted of different types of noise: the probe was a pink noise stimulus [1/f noise: 55 dB(A)] or blue noise stimulus [f noise: 53 dB(A)] and the memory cue was a white noise stimulus [58 dB(A)]. The white noise stimuli that were used in the matching task and the auditory block were the same. The response stimulus in the matching task was a grey horizontal line spanning the width of the screen with a height of 0.1°, with a rectangle (12.2 cd/m^2^, 0.1° x 1.0°) as mouse cursor that could only be moved in the horizontal plane.

### Data analysis

#### Pre-processing and exclusion

All experiments used the same analysis procedure. The eye-movement data was pre-processed with Python 2.7 (the data and analyses are registered on the Open Science Framework website [24]). For each trial, we determined several exclusion criteria. First, we determined whether fixation was established appropriately up until the saccade cue. We excluded a trial if a sample was recorded more than 2.5° away from the fixation point between the onset of the memory cue and the saccade cue (half of the distance between the fixation point and memory cue, see Fig 1C; Experiment 1: 6.2% of all trials, Experiment 2: 4.9% of all trials). This is important, because additional saccades may elicit additional remapping processes, which could affect retinotopic lingering. We also excluded trials based on saccade metrics. Saccades were excluded if not performed within 80 to 1000 ms after the onset of the saccade cue (Experiment 1: 5.3% of all trials, Experiment 2: 4.7% of all trials), or if the amplitude was lower than 8° or higher than 12°, i.e. if the participants overshot or undershot the saccade target (Experiment 1: 7.5% of all trials, Experiment 2: 8.0% of all trials). Lastly, we excluded trials in which the probe was presented during the saccade (Experiment 1: 3.3% of all trials, Experiment 2: 3.3% of all trials). One participant reversed their response during the probe discrimination in the Auditory Remapping block in Experiment 1 (e.g. consistently pressed the key corresponding with ‘Left’ when the answer was ‘Right’). We discovered this during the experiment, and rather than correcting the observer during the experiment (and slowing reaction times), the participant maintained this mapping and we inverted the responses afterwards. In total on average 390 (out of 460, range: 203 to 439) trials remained per participant for analysis in Experiment 1, and 391 trials (out of 480, range: 261 to 463) per participant remained in Experiment 2.

#### Statistical analyses

We first determined whether there was an effect of memory cue location (Fig 1D) with a Bayesian t-test [25]. If not, the left/right cue conditions were collapsed into three probe location conditions (Spatiotopic, Retinotopic, or Neutral). A Bayes Factor (*BF*) of 3 or higher indicates positive evidence in favor of the alternative model (*BF*_*10*_) or in favor of the null-model (*BF*_*01*_) [26]. We analyzed the data with full-factorial linear mixed models. These models predicted reaction time to the probe and included fixed effects for Probe location (3 levels; Spatiotopic, Retinotopic trace, or Neutral), Probe delay (continuous; Experiment 1: ~50 to ~400 ms, Experiment 2: ~30 to ~120 ms) and Task modality (auditory, or visual). Note that we included the offline calculated Probe delay to the analysis, although we specified bins after which the probe was presented, we calculated a more accurate (and continuous) measure of Probe delay for statistical analyses based on offline saccade detection algorithms. A random intercept was added per participant. We compared the full-factorial model to a null model, using a Chi-square test, with a significance criterion of *α* = 0.05. For each parameter in the model, we report the *β*-estimates, standard errors, and *t*-values, using a significance criterion of *α* < 0.05. We used Bayesian t-tests to further describe null-effects of interest (e.g. absence of differences of effects between the auditory and visual task). Note, that the linear mixed effects models are relative to a reference model, which is the Auditory modality, Neutral probe condition with a delay of 0 ms. The figures in the results section are based on statistical comparisons to this reference model.

We analyzed the proportion correct answers to the probe with a full-factorial generalized linear mixed model. This is the same analysis as used for the reaction times, but with a logit link function to better account for ceiling/floor effects. For this analysis, we had to bin the data, due to convergence issues. As the experiment was designed with 50ms (online registered) delay bins in mind, we divided the offline registered (actual) delays into 50 ms bins. For Experiment 2 we binned the data into 30ms bins. This solved convergence issues for both analyses. Note that for the reaction time analyses, we used a continuous measure, and therefore did not need to use bins.

## Results

To investigate whether auditory spatial attention lingers in retinotopic coordinates, thus revealing retinotopic encoding of auditory spatial attention, we ran a similar procedure in both Experiment 1 and Experiment 2. In Experiment 1 we investigated the presence and long-term decay of the auditory spatial attention across an eye-movement. We designed Experiment 2 to investigate whether auditory visual attention is available in retinotopic coordinates *immediately* after a saccade. We only expect to find spatiotopic updating in the visual task, as the lack of visual stimuli in the auditory task may slow spatiotopic updating [23].

### Experiment 1

To recapitulate, we replicated the visual experiment by Golomb and colleagues [17], and further tested an auditory variant of the task. If reference frame transformations occur between visual and auditory spatial input, auditory locations perceived before a saccade should be available in retinotopic coordinates after a saccade. If auditory locations are encoded into retinotopic coordinates, we expected participants to react faster to probes presented after the saccade at the location of the retinotopic trace, regardless of sensory modality.

#### Matching task

**Fig 2:**
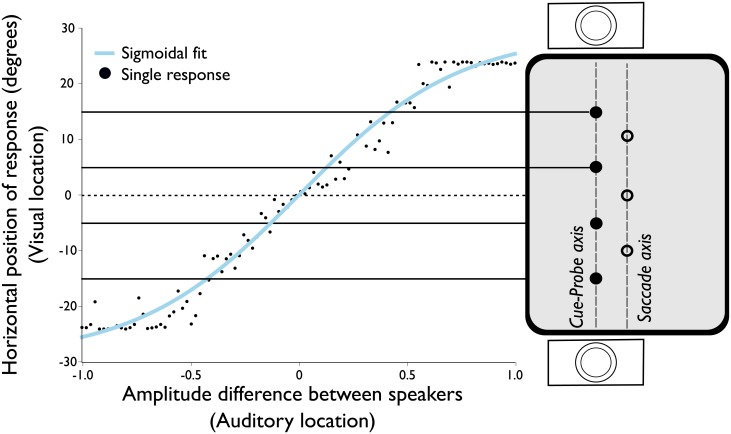
Sigmoid fit of pointing responses to auditory locations of a single participant in the matching task. The black dots show the participant’s localization response to a panned white-noise stimulus. The blue line shows the sigmoid fit to the responses. The locations of the probes used in the auditory block are superimposed on the right side and are connected via a black line. Note that the illustration of the screen is rotated by 90 degrees here, with respect to Fig 1, to align the stimulus location with the vertical axis in the graph.

To match the perceived visual and auditory locations, we ran a matching task (see Fig 1B) for each participant. Each trial, a white-noise stimulus was presented with different panning values between the speakers (see Fig 1A), the participant then clicked the location on the screen that matched the perceived origin of the sound. We fitted a sigmoid relation between speaker panning values and degrees of visual angle on the screen (Fig 2 for an exemplary participant, S1 Fig. for all participant fits). Participants were excluded from further analysis if they did not perceive the most extreme panning values coming from the locations used in the subsequent auditory task (15° from central fixation, Experiment 2: 2 participants). For example, one of the excluded participants in Experiment 2 perceived a panning value of 1 (right speaker full volume) at a location of 5 degrees visual angle, which prohibited us from presenting an auditory probe at 15 degrees visual angle for this participant.

#### Cue/Saccade direction congruency

Before collapsing the probe locations across the levels of the factor Cue location (Fig 1D) we performed a Bayesian t-test. The results show that saccade/cue location congruent trials (e.g. cue right/saccade right, *Med*. = 535.8 ms, *SD* = 179.4 ms) or saccade/cue location incongruent trials (e.g. cue right/saccade left, *Med*. = 531.37 ms, *SD* = 179.6 ms) were not significantly different from one another, *BF*_*01*_ = 32.3, *95%CI* = [-11.48, 11.70]. We collapsed the saccade/cue location conditions, resulting in three main conditions for probe location: Spatiotopic, Retinotopic trace, and Neutral (Fig 1C). The design in the current experiment is somewhat limited in the sense that the retinotopic location is always at a closer eccentricity than the Spatiotopic and Neutral probes, with respect to post-saccadic fixation point, both when participants made a saccade to the left and right. However, we found no effect of saccade direction on reaction time to the Spatiotopic or Neutral probes. Therefore, we assumed that the eccentricity of the probe did not affect reaction times. Lastly, as an additional test we repeated the main linear mixed models without collapsing the probe direction, but instead added probe direction as a random effect to the linear mixed model. Including probe direction in the model did not significantly affect the results. Thus, here we report the models in which we collapsed over cue location below.

#### Response times

To test whether participants (*N* = 17) reacted significantly faster to probes presented at the location of the Retinotopic trace, we constructed a linear mixed model. The full-factorial model included reaction time to probe as the dependent variable, a random intercept per participant and three fixed effects. The fixed effects were: Task Modality (Auditory or Visual), Probe Location (Spatiotopic, Retinotopic trace or Neutral) and Probe Delay (continuous from 50 ms to 400 ms delay between saccade offset and probe onset). The full-factorial linear mixed model outperformed a null model, which only included a random intercept per participant, *ΔBIC* = 384, *Χ*^*2*^(11) = 499.67, *p* < 0.001. Lastly, we investigated the assumption of linearity across delays by fitting a generalized additive mixture model, and found no evidence for non-linearity, *F*(1,3) = 2.05, *p* = 0.10.

#### Responses to retinotopic probes

**Fig 3:**
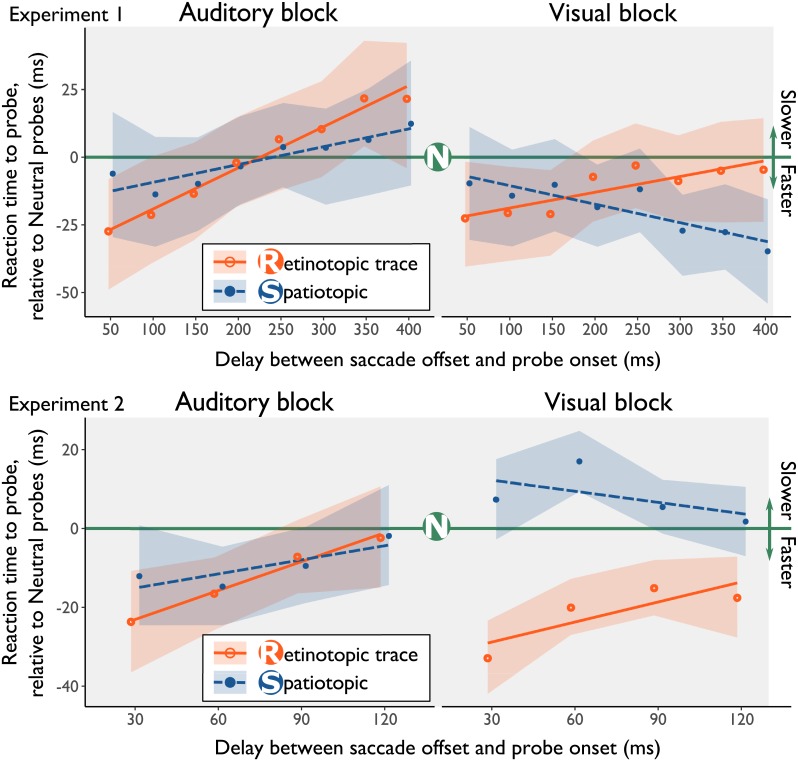
Results from the linear mixed effects models in Experiment 1 and Experiment 2. The green line represents the fit of the linear mixed model of reaction times to probes shown at the neutral location, all other lines are drawn relative to the neutral condition. The lines represented the fits from the linear mixed models, the points indicate the binned average data, after correcting for online saccade detection. In both the visual and auditory experimental block, probes at the location of the retinotopic trace are reacted to significantly faster. This facilitation decreases with longer delays between saccade offset and probe onset, indicating that the retinotopic trace extinguishes over time. Shaded regions represent bootstrapped 95% CI’s. To reduce visual overlap, the orange and blue line have been offset slightly in the horizontal direction.

The full-factorial model (Fig 3, top panels) revealed an offset difference for Probe Location, where participants reacted significantly faster to probes presented at the location of the Retinotopic trace at the earliest delay relative to probes at the Neutral location, *β* = -29.37, *SE* = 14.13, *t* = -2.08, *p =* 0.04. Furthermore, the model showed that the relative facilitation in reaction times to probes presented at the location of the Retinotopic trace diminished over time, *β* = 0.15, *SE* = 0.059, *t* = 2.52, *p* = 0.01. The mean RTs in Experiment 1 across conditions are shown in S1 Table. A comparison between the raw data and the linear mixed effects model is shown in S2 Fig.

#### Responses to spatiotopic probes

The analysis did not provide evidence that probes presented at the Spatiotopic location were reacted to differently compared to the Neutral location, *β* = -8.45, *SE* = 14.02, *t* = -0.60, *p =* 0.54. We also found no evidence that the facilitation at the Spatiotopic location changes with longer delays, *β* = 0.04, *SE* = 0.059, *t* = 0.68, *p =* 0.49. All beta-estimates for the full-factorial model are shown in S2 Table.

#### Comparison effects of visual and auditory retinotopic trace

Averaged across delays, we found no difference in the amount of facilitation of responses to probes presented at the location of the retinotopic trace between the Visual task (*Med*. = -47.6 ms, *SD* = 146.4 ms) and the Auditory task (*Med*. = -49.8 ms, *SD* = 196.50 ms), *BF*_*01*_ = 7.62, *95%CI* = [-4.5, 25.9]. Together, these results indicate that observers reacted faster to probes that were presented at the location of the Retinotopic trace, regardless of their sensory modality.

#### Proportion of answers correct

Lastly, we investigated whether response accuracy differed across locations and/or delays. We used a similar analysis as the analysis used for the reaction times, but with response to the probe (correct/incorrect) as dependent variable. Overall, the accuracy was high (*M*_*correct*_ = 0.96). The analysis showed a significant effect only for Probe delay, where probes presented later after saccade landing were responded to more accurately than earlier delays, *β* = 0.54, *SE* = 0.27, *z* = 2.033, *p* = 0.04. However, this should be interpreted with caution, as the full-factorial model (*BIC* = 2679, *df* = 13) did not outperform a null-model (*BIC* = 2600, *df* = 2, *Χ*^*2*^(11) = 16.66, *p* = 0.12). This is a sign of overfitting, possibly due to many participants performing at ceiling (56 out of 204 proportions correct per condition per participant are at 1.0). In conclusion, we found no evidence of changes in accuracy across different probe locations and task modality.

### Experiment 2

In Experiment 2 (N = 17), we set out to replicate Experiment 1, and investigated whether reaction times to probes at the location of the Retinotopic trace are reacted to faster immediately after the saccade. We designed Experiment 2 so that the offset (i.e. the intercept) of the linear mixed model reflects the first moment after the saccade. After offline encoding of saccades in Experiment 1, we noticed that probes were presented ±30 ms relative to the online definition of a saccade offset. Therefore, we presented the probes 30, 60, 90, or 120 ms after saccade offset (defined online) in Experiment 2 (Fig 1C). After offline saccade detection in Experiment 2, we found that 30 to 42 trials (out of ~990 trials) per condition were trials in which the probe was presented immediately (the first screen update) after the saccade had landed.

#### Reaction times at retinotopic & spatiotopic location

As in Experiment 1, the full-factorial linear mixed model outperformed a null-model, *ΔBIC* = 415, *Χ*^*2*^(11) = 510.41, *p* < 0.001. The beta estimates of the full-factorial model are shown in S4 Table. Participants reacted significantly faster to probes presented at the location of the Retinotopic trace at the earliest delay relative to probes at the Neutral location, *β* = -30.21, *SE* = 13.61, *t* = -2.20, *p* = 0.03 (Fig 1, bottom panels). The reduction of the retinotopic trace over time was not significant (likely due to the smaller timescale of the experiment), *β* = 0.20, *SE* = 0.13, *t* = 1.50, *p* = 0.13. We found no significant evidence for relative facilitation at the Spatiotopic location, *β* = -13.04, *SE* = 13.75, *t* = -0.95, *p* = 0.34, or that facilitation at the Spatiotopic location changed over time, *β* = 0.06, *SE* = 0.13, *t* = 0.48, *p* = 0.63. The means across conditions are shown in S3 Table. The raw data, and the model are shown in S3 Fig.

#### Comparison effects of visual and auditory retinotopic trace

Like Experiment 1, the amount of response facilitation of probes presented at the location of the retinotopic trace was similar for Auditory (*Med*. = -47.4 ms, *SD* = 127.3 ms) and Visual probes (*Med*. = -58.8, ms, *SD* = 177.4 ms), *BF*_*01*_ = 8.66, *95%CI* = [-5.1, 23.3]. These results suggest that attention is present in retinotopic coordinates immediately after a saccade for both auditory and visual stimuli. The magnitude of the attentional benefit is similar for both auditory and visual input.

#### Proportion of answers correct

In Experiment 2, participants gave the correct answer for a large proportion of the trials (*M*_*correct*_ = 0.962). A generalized linear mixed model analysis revealed no effects of Probe delay, Task (A or V), or Probe location. Importantly, the full-factorial model (*BIC* = 1925, *df* = 13) did not outperform a null model (*BIC* = 1844, *df* = 2, *Χ*^*2*^(11) = 14.37, *p* = 0.21), which fits only a random intercept per participant. From this we can conclude that there is no significant difference in accuracy between conditions.

### Combined analyses Experiment 1 and 2

In Experiment 1 and Experiment 2, we found attentional facilitation at retinotopic locations for visual and auditory probes. However, we found no support for spatiotopic updating relative to the control condition (which was observed in prior studies). However, in both experiments a trend was visible in the expected direction: responses to probes at the Visual Spatiotopic location became faster with longer delays relative to the other conditions. As Experiment 1 and Experiment 2 had no overlapping participants and were essentially the same experiment, but with a different range of probe delays, we performed the same analyses on the combined datasets of Experiment 1 and 2. Lastly, in our prior experiments we lacked the power to show differences between retinotopic and spatiotopic conditions, as the difference between these conditions is much lower, and thus requires more power to detect. In the combined dataset we can investigate, exploratively, the difference in facilitation between the spatiotopic and retinotopically presented probes using a sequential analysis. The results from the exploratory analyses were as follows.

#### Response times

Overall, participants reacted faster to probes in the Visual task, *β* = -104.58, *SE* = 8.46, *t* = -12.36, *p* < 0.01, and responses became faster as the delay between saccade offset and probe onset increased, *β* = -0.18, *SE* = 0.03, *t* = -5.57, *p* < 0.01. The decrease in reaction time with increasing delay was more pronounced in the Auditory task, *β* = 0.13, *SE* = 0.05, *t* = 2.91, *p* < 0.01.

The retinotopic results from the analysis are consistent with the results described in Experiment 1 and Experiment 2. The participants reacted faster to probes that were presented at the Retinotopic trace when shown directly after the eye-movement, *β* = -25.65, *SE* = 8.21, *t* = -3.13, *p* < 0.01, and this retinotopic benefit decayed over time, *β* = 0.14, *SE* = 0.05, *t* = 3.11, *p* < 0.01, both with respect to the neutral condition. Lastly, the analysis indicated a 3-way interaction effect (Modality x Delay x Location), where the slope in the Spatiotopic-Visual condition is different relative to the other conditions, *β* = -0.17, *SE* = 0.07, *t* = -2.55, *p* = 0.01. This indicates that over time, responses to Visual-Spatiotopically presented probes get faster over time, providing some (anecdotal) evidence for spatiotopic updating within this paradigm. The Beta estimates for the model are shown in S6 Table. This result provides some evidence for spatiotopic updating, consistent with prior findings, but *only* for the visual modality.

### Proportion correct

Analyzing the proportion of answers correct for the combined dataset did not yield any different results from prior analyses. Comparing a full-factorial linear mixed model (*BIC* = 4481, *df* = 13) to a null-model reveals that the null-model outperforms the full-factorial model (*BIC* = 4403, *df* = 2, *Χ*^*2*^(11) = 24.97, *p* < 0.01). Thus, the accuracy did not differ between the various conditions.

#### Sequential analysis—Retinotopic/Spatiotopic differences

Lastly, we ran a sequential analysis on the combined dataset, as it can provide information on whether effects were not found due to a lack of power. Here, we investigate whether retinotopically presented probes have a different offset, and slope, than spatiotopically presented probes. Rerunning the linear mixed model with the Retinotopic trace condition as the reference indicates that there is a difference in offset between retinotopic and spatiotopic probes, *β* = -15.84, *SE* = 8.00, *t* = -1.98, *p* = 0.04, as well as a slope difference, *β* = -0.09, *SE* = 0.04, *t* = -2.08, *p* = 0.03.

The results of the sequential analysis are shown in S4 Fig. (with the Neutral condition as the baseline) and S5 Fig. (with the Retinotopic condition as the baseline). It seems that our assertion that we lacked power in the single dataset was correct, as differences between Retinotopic and Spatiotopic conditions are only statistically significant at N = 27 (for a difference in offset) and at N = 26 (for a difference in slope), and remain significant with further inclusion. Note that these results are tenuous, as we did not design the experiment or our analyses to (initially) test for a difference between spatiotopically and retinotopically presented probes.

Lastly, we compared our data to the raw data reported in Golomb and colleagues’ 2008 study [17], as provided in the supplement to the article. To this end, we only used the data from the visual task in Experiment 1 and Experiment 2. Much like our study, Golomb ran an experiment with longer delays (delay bins of 75ms, 150ms, 250ms, 400ms, and 600 ms), and an experiment with shorter delays (delay bins of 0ms, 25ms, 50ms, and 75ms). For these comparisons, we recoded our offline corrected delays in the bins used by Golomb and colleagues [17], by rounding the delay to the nearest bin. First, we compared the standard errors of the mean in Experiment 1 to the grand means reported by Golomb, et al. [17], and find that 8 out of 12 data points lie within the standard error of our data. Next, we compared the standard error of the mean in Experiment 2 to the grand means of the experiment with shorter delays. Here we find that 9 out of 12 data points lie within the standard error of our data. This comparison suggests that the data in our study and prior literature is quite similar. The comparison is visualized in S6 Fig.

## Experiment 3

In Experiment 1 and 2 we have demonstrated that both auditory and visually evoked spatial attention lingers at the retinotopic coordinates after a saccade, facilitating responses to auditory and visual probes presented at that location. We reasoned that retinotopic encoding of non-visual (e.g. auditory) locations may contribute to multisensory perceptual stability in the face of frequent disruptions of visual processing due to saccades. However, one thing remains unclear given the current results.

First, it is unclear whether retinotopic encoding of spatial attention is specific to one modality (i.e. intra-modal) or shared between modalities (i.e. cross-modal). That is, visual and auditory spatial attention could be independently encoded into retinotopic coordinates or retinotopic encoding of spatial attention could be cross-modal. We expected some form of peri-saccadic cross-modal attentional orienting to occur, as cross-modal cueing effects have been readily observed in many cross-modal cuing tasks [27–30]. Studies on cross-modal cueing have shown that without spatial relevance, auditory information can be processed tonotopically, resulting in a lack of spatial cueing effects [27,28,31]. This is in contrast with visual spatial cuing effects, which are inherently spatial due to the retinotopic organization of the visual processing hierarchy. Prior research has shown that cross-modal spatial attentional cueing can be asymmetric (A-V cuing, but not V-A cuing) under certain circumstances [27,28,31], and that cross-modal cueing effects are only present in both directions across sensory modalities when the spatial location of auditory stimuli is task-relevant [27,28,31]. Running a cross-modal variant of our paradigm allows us (1) to investigate whether retinotopic encoding is modality specific, and (2) to investigate the role of spatial relevance for responses to auditory stimuli at prior retinotopic coordinates.

In Experiment 3, we ran a cross-modal variant of the task used in Experiment 1 and Experiment 2. To elaborate, the paradigm used in all experiments consisted of two tasks, a spatially relevant memory cue task, and a spatially irrelevant probe discrimination task. In Experiment 1 and Experiment 2, participants completed a fully unimodal Auditory cue/Auditory probe and a unimodal Visual cue/Visual probe task. In Experiment 3, participants completed an Auditory cue/Visual probe task and a Visual cue/Auditory probe task. If retinotopic deployment of attention occurs separately for auditorily evoked and visually evoked spatial attention, then there should be no retinotopic effects in either cross-modal task. That is, auditory evoked retinotopic attention only effects auditory processing of stimuli at that location and the same for visual evoked retinotopic attention. Next, if retinotopic deployment of auditory spatial attention requires spatial relevance, an asymmetry between the two cross-modal cueing tasks would be observed. In Experiment 3, auditory information was only spatially relevant in the Auditory cue/Visual probe condition, as participants remembered and reported the location of the auditory memory cue, whereas the visual probe is implicitly spatially relevant. In the Visual cue/Auditory probe condition the auditory task was a spatially irrelevant frequency discrimination task. Given the large body of literature on cross-modal cueing effects, we expected to find evidence of cross-modal retinotopic attentional facilitation in at least the task with auditory spatial relevance (Auditory cue/Visual probe) [27,28,31].

### Methods

#### Subjects and procedure

The subjects (*N* = 20, 18 Female, *M*_*age*_ = 20.2) completed 252 trials in the Auditory cue/Visual Probe task and 252 trials in the Visual cue/Auditory probe task. Half of the participants completed the Auditory cue/Visual probe before completing the Visual cue/Auditory probe task. This order was reversed for the remaining half. Participants found the task noticeably harder than the task in Experiment 1 and Experiment 2. We found a higher rate of exclusion in this experiment, as compared to Experiment 1 and Experiment 2, after examining the quality of the data when we tested 12 participants. Therefore, after testing 12 of the subjects in Experiment 3, we chose to test up to 20 subjects.

Importantly, in Experiment 3 the modalities of the cue and the probe differ (cross-modal cueing). To recapitulate, the cue task required observers to maintain a location in memory across saccades, which creates a framework for spatial relevance. The probe task requires observers to react to the identity of the probe as quickly as possible (and the location is incidental). Although tasks are the same in task demands, we expect an asymmetric cueing effect driven by the differences in the spatial properties of the task. We expect cueing effects even when the visual spatial locations are not relevant (Auditory cue/Visual probe task). If retinotopic encoding of auditory spatial attention only occurs with spatial relevance we expected to replicate retinotopic attentional facilitation only for the Auditory cue/Visual probe task, but not for the Visual cue/Auditory probe.

Based on the results from Experiment 1 and Experiment 2 we changed the timing of the probe presentation. In Experiment 3 the probe was presented either 30 ms, 120 ms or 210 ms after saccade offset. We determined that this would give a good estimate of the attentional facilitation both directly after the saccade (at ~30 ms) as well as the decay of retinotopic lingering at longer delays (at >200 ms).

#### Data exclusion

The exclusion criteria were identical to the criteria in Experiment 1 and Experiment 2. We excluded a trial if a sample was recorded more than 2.5° away from the fixation point between the onset of the memory cue and the saccade cue (12% of all trials). Trials were excluded if saccades were not performed within 80 to 1000 ms after the onset of the saccade cue (8% of all trials), or if the saccade amplitude was not between 8° and 12° (20% of all trials). Lastly, we excluded trials in which the probe was presented during the saccade (5% of all trials). After exclusion 6914 trials were left in total (69% of all trials), on average 345 trials per participant (out of 504 trials, range 110 to 465). The statistical analyses were identical to the previous experiments.

### Results

#### Reaction times at retinotopic/spatiotopic location

**Fig 4:**
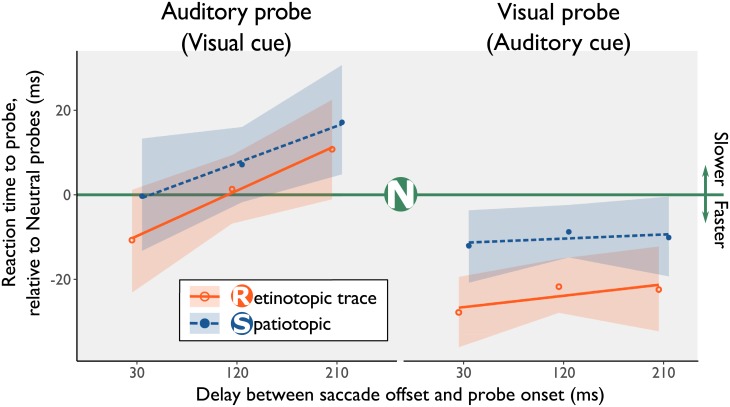
Results from the linear mixed effects model in Experiment 3. The green line represents reaction times to probes shown at the neutral location, all other lines are drawn relative to the neutral condition. The lines represented the fits from the linear mixed models, the points indicate the binned average data, after correcting for online saccade detection. In both the visual and auditory experimental block, probes at the location of the retinotopic trace are reacted to significantly faster. Shaded regions represent bootstrapped 95% CI’s. To reduce visual overlap, the orange and blue line have been offset slightly in the horizontal direction.

The full-factorial linear mixed model, containing the independent variables for Task modality (A cue/V probe or V cue/A probe) and Probe Location (Neutral, Spatiotopic, or Retinotopic) and a random intercept per participant (*BIC* = -159, *df* = 14), outperformed a null model with only a random intercept per participant (*BIC* = 88441, *df* = 3, *Χ*^*2*^(11) = 88698, *p* < 0.01). The full-factorial linear mixed model analysis (Fig 4) again showed evidence for facilitation at the location of the retinotopic trace relative to neutral probes, *β* = -49.7, *SE* = 20.1, *t* = -2.48, *p* = 0.01. Similar to Experiment 1 and Experiment 2, if the delay increased between saccade onset an probe onset participants responded faster, *β* = 0.346, *SE* = 0.092, *t* = -3.46, *p* < 0.01. We found the participants responded slower in the Visual cue/Auditory probe task relative to the Auditory Cue/Visual Probe task, *β* = 164, *SE* = 19.9, *t* = 8.23, *p* < 0.01. All other fixed effects were not statistically significant, *t* < 1.12, *p* > 0.26, indicating no evidence for (slowed) spatiotopic updating. Condition means are shown in S5 Table. The raw data, and the bootstrapped estimates from the linear mixed effects model are shown in S7 Fig.

In Experiment 1 and Experiment 2, the magnitude of attentional facilitation at the location of the retinotopic trace was similar for the auditory and visual sensory modality. In Experiment 3, however, there was a clear difference in the magnitude of retinotopic attentional facilitation between tasks (A-V vs. V-A), *BF*_*10*_ = 157, *95%CI* = 12.1 to 36. A Bayesian t-test (one for each task), indicated a null effect of attentional facilitation at the Retinotopic trace location in the Visual cue/Auditory probe condition, *BF*_*01*_ = 30.7, *95%CI* = -11.4 to 12.6. In contrast, in the Auditory Cue/Visual Probe condition we found strong evidence for attentional facilitation at the Retinotopic trace location, *BF*_*10*_ = 801,223, *95%CI* = -31.0 to -16.7.

In sum, we observed cross-modal attentional facilitation at the location of the retinotopic trace in the Auditory Cue/Visual Probe condition, but not in the Visual cue/Auditory probe condition. Thus, in line with previous research on cross-modal spatial attention, cross-modal attentional facilitation at the location of the retinotopic trace is only present when auditory spatial information is task-relevant.

#### Accuracy at retinotopic/spatiotopic location

Overall, the accuracy was very high (*M*_*correct*_ = 0.92). Analyzing the accuracy yielded similar results to prior accuracy analyses. Comparing a full-factorial linear mixed model (*BIC* = 3782, *df* = 13) to a null-model reveals that the null-model outperforms the full-factorial model (*BIC* = 3739, *df* = 2, *Χ*^*2*^ = 54.48, *p* < 0.01). We conclude that, in Experiment 3 there was no difference in the proportion of correct answers.

## General discussion

In the current study, we demonstrated that auditory spatial attention is encoded in a retinotopic reference frame across eye-movements. Retinotopic encoding of spatial attention facilitates both auditory and visual information processing at the location of the retinotopic trace. Our findings are in line with the studies by Golomb and colleagues who showed that after a saccade visual spatial attention lingers in retinotopic coordinates, and that visual attention slowly updates to the spatiotopic location [17,23,32]. Our study expands these findings by revealing the multisensory/cross-modal nature of retinotopic lingering of spatial attention across eye-movements.

Prior studies have shown that visual attention is retinotopically encoded and lingers in retinotopic coordinates after a saccade [17,23,32]. We hypothesized that auditory spatial attention may be retinotopically encoded around the time of a saccade as well. We found that spatial pointers obtained via both vision and audition elicit a retinotopic trace, immediately after a saccade. The retinotopic lingering of auditory evoked spatial attention directly after a saccade indicates that auditory information is encoded in retinotopic coordinates around the time of a saccade. Interestingly, we found that the attentional benefit at the location of the retinotopic trace (shorter reaction time) was similar across modalities. This similarity of attentional benefits across modalities suggests a common attentional updating mechanism shared between modalities (around the time of a saccade).

Furthermore, we observed that auditory evoked spatial attention elicited visual retinotopic effects, but not the other way around. This asymmetry suggests that auditory spatial attention does not mandatorily linger in retinotopic coordinates around the time of a saccade. Asymmetries in cross-modal cueing effects were found in prior studies as well, where cross-modal spatial attentional effects are only present when auditory spatial locations are made task-relevant [27,33,34]. When responses to auditory targets rely on frequency discrimination, responses can be based on tonotopic representations of the auditory target, which lack spatial specificity (although spatial information is processed as well) [27,33,34]. This is in contrast with visual representations, which are inherently spatial due to the retinotopic organization of the early visual processing areas. In studies by Ward and colleagues [28,31], cross-modal cueing effects were only present in an auditory cue-visual probe task, but not in a visual cue-auditory probe task. This asymmetry was not observed when auditory spatial information was made task-relevant [28,35]. Our study also demonstrates that auditory spatial attention is likely only deployed when auditory space is task-relevant.

For auditory information to be retinotopically encoded, a reference frame transformation is required from craniotopic auditory coordinates to retinotopic auditory coordinates. Studies using animal models have provided insight into the neurophysiological underpinnings of reference frame transformations in the midbrain. Broadly speaking, the location of visual input is represented in a retinotopic reference frame within the superior colliculus [36,37], whereas auditory input is represented in a head-centered reference frame in the inferior colliculus [38–40]. Auditory localization is based on multiple binaural and monaural cues, including differences in intensity and arrival time between the ears (binaural cues) for horizontal sound localization [41]. The binaural cues for horizontal localization are amplitude differences of the sound wave when it reaches each ear (interaural level differences), and phase differences of sound waves due to differences in arrival times to the ears (interaural time differences). Binaural cues are processed in parallel in brain stem pathways, converging in frequency tuned maps in the central nucleus of the inferior colliculus [38,39]. The information across the frequency tuned maps for vertical and horizontal localization are integrated into a single, three-dimensional, auditory map in the external nucleus of the inferior colliculus [38,39,42]. This auditory map is consequently relayed and integrated with a retinotopic visual map in the superior colliculus, resulting in a multimodal, retinotopic map of space [10,39]. Additionally, gaze control circuitry in the forebrain have been identified to modulate auditory responses in the midbrain, showing direct connectivity between higher order visual areas and structures such as the inferior colliculus [43]. These processes have been studied extensively in animal models and have been corroborated by results from studies with human subjects, for example, by measuring auditory brainstem responses to auditory stimuli [44–46]. The role of the superior colliculus has therefore been described as unifying attentive and orienting behavior between senses, as sensory maps in the superior colliculus share a similar axis system [3]. It seems that the pathways between the brain stem, inferior colliculus, and superior colliculus may play a critical role in the subjective experience of sensory unity by integrating audiovisual location information in shared (retinotopic) reference frames. Our results reveal that reference frame transformations are relevant to human perception around the time of a saccade. Likely, reference frame transformations allow humans to quickly compare locations of sensory input regardless of differences in coordinate systems native to the modality.

In addition to retinotopic facilitation effects, we observed spatiotopic updating in the visual condition. However, we did not find support for spatiotopic updating in the auditory condition in Experiment 1 and 2. We designed Experiment 3 only with retinotopic effects in mind, not expecting to find cross-modal spatiotopic updating, which limits the conclusions that can be drawn about spatiotopic updating based on the results in that experiment. As previously shown [23], spatiotopic updating may be affected both by the amount of visual stimuli (being slowed by less visual stimulation), and task instruction (being attenuated when participants are instructed to disregard spatiotopic coordinates).

In Experiment 1 of the current study, auditory spatial attention did not update faster to spatiotopically presented probes when compared to auditory neutral probes. When investigating the results from the visual task, we did observe attentional updating at spatiotopic locations when we combined the data from both Experiment 1 and Experiment 2. This discrepancy between visual and auditory spatiotopic updating is unsurprising. First, spatiotopic updating of attentional facilitation becomes less apparent if participants remember locations in retinotopic coordinates, whereas retinotopic facilitation is present without explicit task-relevance [17]. We explicitly chose not to instruct participants which coordinate system they should use, to not bias participants to remember auditory probes in retinotopic coordinates only. We postulate that by not instructing participants to update auditory probes in spatiotopic coordinates they were not focusing on spatiotopic locations. Secondly, work by Golomb and colleagues has shown that spatiotopic updating is facilitated (sped up) by providing more visual input (a grid overlaying the background [23]). Conversely, removing visual input may slow spatiotopic updating. In the current study, in the visual conditions, cues and probes may have provided a spatial framework, allowing for spatiotopic updating. Therefore, the lack of spatiotopic updating in the auditory task in the current study is inherently confounded by the lower amount of visual stimulation in the auditory block, which in turn may further slow the spatiotopic updating of auditory spatial attention.

Finally, we would like to note that auditory information is not actually represented in retinotopic coordinates, but rather in *oculocentric* coordinates. This distinction is important, as auditory stimuli are not projected onto the retina. However, we chose to keep the terminology consistent with the studies of Golomb and colleagues [17], as we consider this study to be an extension of the literature on the retinotopic trace, and spatiotopic updating.

The current series of experiments shows that auditory information is, likely, pre-saccadically encoded into retinotopic coordinates, causing post-saccadic lingering of the retinotopic trace for auditory input. Encoding auditory spatial attention in a retinotopic reference frame may facilitate comparing auditory and visual spatial information, allowing for spatial alignment of these sensory systems, and intramodal and cross-modal attentional facilitation in humans.

## Supporting information

S1 Fig Sigmoid fit of pointing responses to auditory locations of all participants in the matching task. The black dots show individual responses. The cyan colored lines represent the sigmoid fit used to match visual and auditory location.
(EPS)

S2 Fig Overview of all data in Experiment 1. The colored squares are counts in a 2D histogram. The horizontal bins in the histogram are 50 ms wide, the vertical bins are 30 ms wide. The median, 1^st^ and 3^rd^ quartile and estimates from the linear mixed model are plotted per condition.
(EPS)

S3 Fig Overview of all data in Experiment 2. The colored squares are counts in a 2D histogram. The horizontal and vertical bins in the histogram are 30 ms wide. The median, 1^st^ and 3^rd^ quartile and estimates from the linear mixed model are plotted per condition.
(EPS)

S4 Fig Sequential analysis using Neutral locations as reference. Each dot represents one linear mixed model analysis, the x-axis shows the number of participants included in the analysis (in no particular order) and the y-axis shows the p value for the parameter. Black dots indicate a value of p < 0.05. The line is a log-linear fit to the data and only serves as visual reference. Note that under the null-hypothesis, the p-value is expected to do a random walk.
(EPS)

S5 Fig Sequential analysis using retinotopic trace locations as reference. Each dot represents one linear mixed model analysis, the x-axis shows the number of participants included in the analysis (in no particular order) and the y-axis shows the p value for the parameter. Black dots indicate a value of p < 0.05. The line is a log-linear fit to the data and only serves as visual reference. Note that under the null-hypothesis, the p-value is expected to do a random walk.
(EPS)

S6 Fig Comparison between the current study and the grand means previously reported by Golomb and colleagues’ study. The squares show the grand means of prior literature [17], the diagonal lines and shaded regions show the fitted linear mixed model and standard error on the model. The vertical lines represent the standard error of the mean on our binned data.
(EPS)

S7 Fig Overview of all data in Experiment 3. The colored squares are counts in a 2D histogram. The horizontal bins in the histogram are 90 ms wide, the vertical bins are 30 ms wide. The median, 1^st^ and 3^rd^ quartile and estimates from the linear mixed model are plotted per condition.
(EPS)

S1 Table Table of grand means for Experiment 1. (PDF)

S2 Table Beta estimates, standard error and t values per factor of the full-factorial model specified in Experiment 1. (PDF)

S3 Table Table of grand means for Experiment 2. (PDF)

S4 Table Beta estimates, standard error and t values per factor of the full-factorial model specified in Experiment 2. (PDF)

S5 Table Table of grand means for Experiment 3. (PDF)

S6 Table Beta estimates, standard error and t values per factor of the full-factorial model specified for the combined analysis of Experiment 1 and Experiment 2. (PDF)
