# Supplementary material for: Auditory spatial attention is encoded in a retinotopic reference frame across eye-movements
Source: PLoS One. 2018 Aug 20;13(8):e0202414. doi: 10.1371/journal.pone.0202414 (PMC6101386; doi:10.1371/journal.pone.0202414)
Supplement: S1 Table — (PDF) [file pone.0202414.s008.pdf]

Probe Location	Modality	Online Delay *	# of Trials*	Mean RT (ms)	SD RT (ms)	95% CI
**Neutral**	**Auditory**	50	139	656.80	184.87	31.00
		100	138	643.31	192.80	32.45
		150	141	622.80	196.16	32.66
		200	136	632.94	201.94	34.25
		250	137	608.92	183.69	31.03
		300	135	587.42	188.97	32.17
		350	128	584.46	166.05	29.04
		400	96	590.36	170.38	34.52
	**Visual**	50	124	556.06	114.02	20.27
		100	125	539.37	151.42	26.81
		150	127	546.67	149.78	26.30
		200	139	524.73	137.29	23.03
		250	121	532.06	169.69	30.54
		300	124	510.34	138.47	24.61
		350	114	543.75	149.88	27.81
		400	93	546.23	128.82	26.53
**Retinotopic**	**Auditory**	50	133	637.55	169.24	29.03
		100	149	638.00	210.77	34.12
		150	144	622.96	171.67	28.28
		200	145	618.09	200.32	32.88
		250	136	615.20	207.31	35.16
		300	140	603.12	204.40	34.16
		350	129	599.70	171.87	29.94
		400	92	615.23	204.42	42.33
	**Visual**	50	109	537.64	124.88	23.71
		100	123	535.54	128.41	22.92
		150	124	502.35	139.51	24.80
		200	135	533.01	146.70	24.97
		250	126	508.52	140.04	24.69
		300	134	516.05	130.34	22.27
		350	116	528.95	160.93	29.60
		400	91	548.97	164.53	34.27
**Spatiotopic**	**Auditory**	50	141	662.61	188.92	31.45

	100	138	658.07	217.47	36.61
	150	140	622.21	201.49	33.67
	200	133	581.93	177.88	30.51
	250	142	621.78	179.26	29.74
	300	141	591.80	169.06	28.15
	350	131	611.96	195.84	33.85
	400	98	568.09	178.85	35.86
**Visual**	50	130	542.93	146.49	25.42
	100	126	537.91	170.04	29.98
	150	129	512.68	145.23	25.30
	200	124	511.28	150.47	26.75
	250	122	511.75	163.31	29.27
	300	129	515.73	132.46	23.08
	350	118	503.17	136.13	24.82
	400	87	506.80	128.18	27.32

* We calculated the offline delay, which differed from the online delay. For analyses we used the offline delay. For purposes of this table, we rebinned the offline determined delays back into 50 ms bins. The number of trials reflects the amount of trials after rebinning the conditions, thus the mean RTs approximate the values used in the analyses.
