# Supplementary material for: Auditory spatial attention is encoded in a retinotopic reference frame across eye-movements
Source: PLoS One. 2018 Aug 20;13(8):e0202414. doi: 10.1371/journal.pone.0202414 (PMC6101386; doi:10.1371/journal.pone.0202414)
Supplement: S2 Table — (PDF) [file pone.0202414.s009.pdf]

Factor name	*β*-estimate	Standard error	*t*-value
**Intercept**	658.63	19.88	33.12 *
**Location – Retinotopic trace**	-29.37	14.13	-2.08 *
**Location – Spatiotopic**	-8.45	14.02	-0.60
**Task – Visual**	-110.21	14.44	-7.63 *
**Probe delay**	-0.19	0.04	-4.51 *
**Location – Retinotopic trace * Task – Visual**	13.42	20.55	0.65
**Location – Spatiotopic * Task – Visual**	3.42	20.27	0.86
**Location – Retinotopic trace * Probe delay**	0.15	0.06	2.53 *
**Location – Spatiotopic * Probe delay**	0.04	0.06	0.68
**Task – Visual * Probe delay**	0.16	0.06	2.59 *
**Location – Retinotopic trace * Task – Visual * Probe delay**	-0.13	0.09	-1.48
**Location – Spatiotopic * Task – Visual * Probe delay**	-0.11	0.09	-1.29
