# Supplementary material for: Auditory spatial attention is encoded in a retinotopic reference frame across eye-movements
Source: PLoS One. 2018 Aug 20;13(8):e0202414. doi: 10.1371/journal.pone.0202414 (PMC6101386; doi:10.1371/journal.pone.0202414)
Supplement: S3 Table — (PDF) [file pone.0202414.s010.pdf]

Probe Location	Modality	Online Delay*	# of Trials*	Mean RT (ms)	SD RT (ms)	95% CI
**Neutral**	**Auditory**	30	288	579.81	176.14	20.43
		60	291	609.10	199.09	22.97
		90	271	582.64	180.19	21.55
		120	298	578.27	192.30	21.92
	**Visual**	30	259	525.55	143.97	17.62
		60	250	491.56	118.39	14.75
		90	263	496.82	147.10	17.86
		120	249	480.31	134.71	16.81
**Retinotopic**	**Auditory**	30	271	586.41	168.70	20.18
		60	274	586.36	188.51	22.42
		90	285	563.94	161.67	18.85
		120	286	574.40	186.02	21.65
	**Visual**	30	244	480.05	111.44	14.05
		60	252	477.47	136.18	16.89
		90	242	476.46	147.25	18.64
		120	252	469.75	142.68	17.70
**Spatiotopic**	**Auditory**	30	278	588.91	180.28	21.28
		60	293	571.03	166.37	19.12
		90	290	577.91	192.08	22.20
		120	296	576.23	181.16	20.72
	**Visual**	30	248	515.73	137.65	17.2
		60	255	511.20	145.80	17.98
		90	256	497.05	132.70	16.33
		120	245	487.29	137.56	17.31

* We calculated the offline delay, which differed from the online delay. For analyses we used the offline delay. For purposes of this table, we rebinned the offline determined delays back into 30 ms bins. The number of trials reflects the amount of trials after rebinning the conditions, thus the mean RTs approximate the values used in the analyses.
