# Supplementary material for: Auditory spatial attention is encoded in a retinotopic reference frame across eye-movements
Source: PLoS One. 2018 Aug 20;13(8):e0202414. doi: 10.1371/journal.pone.0202414 (PMC6101386; doi:10.1371/journal.pone.0202414)
Supplement: S4 Table — (PDF) [file pone.0202414.s011.pdf]

Factor name	*β*-estimate	Standard error	*t*-value
**Intercept**	630.27	25.6	24.5 *
**Location – Retinotopic trace**	-30.21	13.61	-2.20 *
**Location – Spatiotopic**	-13.05	13.74	-0.95
**Task – Visual**	-97.13	14.32	-6.78 *
**Probe delay**	-0.25	0.09	-2.72 *
**Location – Retinotopic trace * Task – Visual**	-5.97	20.49	-0.29
**Location – Spatiotopic * Task – Visual**	31.81	20.34	1.56
**Location – Retinotopic trace * Probe delay**	0.20	0.13	1.50
**Location – Spatiotopic * Probe delay**	0.06	0.13	1.50
**Task – Visual * Probe delay**	0.06	0.14	0.48
**Location – Retinotopic trace * Task – Visual * Probe delay**	-0.06	0.19	-0.30
**Location – Spatiotopic * Task – Visual * Probe delay**	-0.21	0.19	-1.07
