# Supplementary material for: Auditory spatial attention is encoded in a retinotopic reference frame across eye-movements
Source: PLoS One. 2018 Aug 20;13(8):e0202414. doi: 10.1371/journal.pone.0202414 (PMC6101386; doi:10.1371/journal.pone.0202414)
Supplement: S5 Table — (PDF) [file pone.0202414.s012.pdf]

Probe Location	Modality	Online Delay*	# of Trials*	Mean RT (ms)	SD RT (ms)	95% CI
**Neutral**	**Auditory**	30	388	598.82	168.01	16.77
		120	414	557.43	156.59	15.13
		210	405	545.22	165.07	16.12
	**Visual**	30	344	516.58	121.67	12.90
		120	373	493.63	125.60	12.79
		210	352	483.29	126.97	13.31
**Retinotopic**	**Auditory**	30	400	585.12	148.80	14.63
		120	405	570.26	179.57	17.54
		210	413	547.07	160.18	15.49
	**Visual**	30	365	490.01	109.50	11.27
		120	350	471.37	125.38	13.18
		210	370	460.65	120.67	12.34
**Spatiotopic**	**Auditory**	30	391	604.41	171.40	17.04
		120	410	564.23	152.62	14.82
		210	406	557.75	162.36	15.84
	**Visual**	30	362	501.81	105.25	10.88
		120	379	482.42	127.23	12.85
		210	387	478.77	121.94	12.19

* We calculated the offline delay, which differed from the online delay. For analyses we used the offline delay. For purposes of this table, we rebinned the offline determined delays back into 50 ms bins. The number of trials reflects the amount of trials after rebinning the conditions, thus the mean RTs approximate the values used in the analyses.
