# Supplementary material for: Auditory spatial attention is encoded in a retinotopic reference frame across eye-movements
Source: PLoS One. 2018 Aug 20;13(8):e0202414. doi: 10.1371/journal.pone.0202414 (PMC6101386; doi:10.1371/journal.pone.0202414)
Supplement: S6 Table — (PDF) [file pone.0202414.s013.pdf]

Factor name	*β*-estimate	Standard error	*t*-value
**Intercept**	642.61	16.03	40.09*
**Location – Retinotopic trace**	-25.65	8.21	-3.13*
**Location – Spatiotopic**	-9.35	8.18	-1.14
**Task – Visual**	-104.58	8.46	-12.36*
**Probe delay**	-0.18	0.03	-5.57*
**Location – Retinotopic trace * Task – Visual**	-0.96	12.04	-0.08
**Location – Spatiotopic * Task – Visual**	20.74	11.96	1.74
**Location – Retinotopic trace * Probe delay**	0.14	0.05	3.11*
**Location – Spatiotopic * Probe delay**	0.04	0.04	0.94
**Task – Visual * Probe delay**	0.13	0.05	2.91*
**Location – Retinotopic trace * Task – Visual * Probe delay**	-0.07	0.07	-1.22
**Location – Spatiotopic * Task – Visual * Probe delay**	-0.17	0.07	-2.55*
